# The Effects of Post‐Activation Performance Enhancement on High Intensity Interval Training: A Comparison of Traditional and Cluster Set Protocols

**DOI:** 10.1002/ejsc.70152

**Published:** 2026-03-05

**Authors:** Merve Cin, Özcan Saygın, Refik Çabuk

**Affiliations:** ^1^ Department of Social Sciences Faculty of Security Sciences Gendarmerie and Coast Guard Academy Ankara Türkiye; ^2^ Department of Coaching Education Yaşar Doğu Faculty of Sport Sciences Muğla Sıtkı Koçman University Muğla Türkiye; ^3^ Department of Coaching Education Yaşar Doğu Faculty of Sport Sciences Ondokuz Mayıs University Samsun Türkiye; ^4^ College of Health and Life Sciences Hamad Bin Khalifa University Doha Qatar

**Keywords:** conditioning activity, conventional set, endurance, intra‐set rest, maximal oxygen uptake

## Abstract

The present study aimed to examine the effects of post‐activation performance enhancement (PAPE) protocols employing half‐squat exercises, performed using either a traditional set (PAPE_traditionalset_) or a cluster set (PAPE_clusterset_), on peak oxygen uptake ($\dot{V}$O_2peak_), the time spent at ≥ 90% maximal oxygen uptake ($\dot{V}$O_2max_), and total exercise duration during high‐intensity interval training (HIIT) in trained athletes. Ten well‐trained endurance athletes involved in endurance sport ($\dot{V}$O_2max_: 63.3 ± 5.1 mL·kg^‐1^∙min^‐1^) completed three HIIT sessions (until failure x (1‐min run at $\dot{V}$O_2max_ velocity + 1‐min active recovery at 30% $\dot{V}$O_2max_ velocity)) under different conditions: (i) control (warm‐up only: jogging and stretching), (ii) warm‐up + three sets of six repetitions of the half‐squat (traditional set; 120 s of rest between sets), and (iii) warm‐up + three sets of 2 + 2 + 2 repetitions of half‐squat (cluster set; 20‐s rest after each two‐repetition cluster and 80‐s rest between sets). No significant differences were found in $\dot{V}$O_2peak_ responses among the three HIIT protocols (control: 62.3 ± 5.1 mL·kg^‐1^·min^‐1^; PAPE_traditionalset_: 61.6 ± 4.0 mL·kg^‐1^·min^‐1^; PAPE_clusterset_: 61.9 ± 3.7 mL·kg^‐1^·min^‐1^; *p* = 0.198). However, both PAPE protocols increased time at ≥ 90% $\dot{V}$O_2max_ (control: 6.21 ± 0.9 min; PAPE_traditionalset_: 7.43 ± 1.2 min; PAPE_clusterset_: 8.11 ± 1.4 min; *p* < 0.001) and total work duration (control: 12.0 ± 0.96 min; PAPE_traditionalset_: 13.4 ± 1.54 min; PAPE_clusterset_: 14.4 ± 1.53 min; *p* < 0.001) compared with control. Additionally, the PAPE_clusterset_ resulted in a significantly longer time spent at ≥ 90% $\dot{V}$O_2max_ (*p* = 0.005) and total work duration compared with the PAPE_traditionalset_ (*p* = 0.043). PAPE protocols, especially using cluster sets, increase time ≥ 90% $\dot{V}$O_2max_ and total work duration during HIIT, potentially enhancing endurance performance.

## Highlights

- Post‐activation performance enhancement (PAPE) protocols incorporating resistance exercises before HIIT session increase total work duration and time spent at ≥ 90% maximal oxygen uptake without altering peak oxygen uptake, maximal blood lactate, or perceived exertion level in well‐trained endurance athletes.
- The ergogenic effects of PAPE during HIIT appear to be driven by improved exercise tolerance and altered $\dot{V}$O_2_ kinetics rather than changes in maximal cardiorespiratory responses.

## 1 Introduction

Performing exercise at intensities close to maximal oxygen uptake ($\dot{V}$O_2max_) imposes significant stress on the body's oxygen transport and utilization systems, thereby playing a crucial role in enhancing endurance performance (Buchheit and Laursen 2013; Midgley and McNaughton 2006). One of the training methods that allows athletes to extend the duration of high intensity exercise is high‐intensity interval training (HIIT) (Buchheit and Laursen 2013; Midgley and McNaughton 2006). During an HIIT session, the percentage of $\dot{V}$O_2max_ achieved and the cumulative time spent at ≥ 90% $\dot{V}$O_2max_ are considered key factors in enhancing the central and peripheral adaptations that improve $\dot{V}$O_2max_ and endurance performance (Buchheit and Laursen 2013; Laursen and Jenkins 2002). Therefore, these two acute responses are regarded as critical stimuli for evaluating the effectiveness of HIIT (Çabuk and Alp 2025; Norouzi et al. 2023; Odden et al. 2024; Thevenet et al. 2007). Although it is well‐known that acute exercise responses do not always accurately reflect the magnitude of adaptations following a training intervention (Odden et al. 2024), Odden et al. (2024) demonstrated that HIIT protocols involving longer cumulative durations at ≥ 90% $\dot{V}$O_2max_ and higher %$\dot{V}$O_2max_ responses yield superior physiological adaptations underlying endurance performance improvements. Recently, studies investigating the optimization of interval training variables to maximize the time spent at ≥ 90% $\dot{V}$O_2max_ have increased (Almquist et al. 2020; Bossi et al. 2020; Held et al. 2023; Rønnestad et al. 2017). Innovative HIIT methods have also been developed to enhance these responses (Çabuk and Alp 2025); however, research on additional strategies remains limited (de Freitas et al. 2019; Pugh et al. 2024; Smith et al. 2022). Including a short‐duration, high‐intensity conditioning activity (e.g., plyometric or moderate‐intensity resistance exercise) immediately before the target effort in warm‐up routines may enhance anaerobic performance components such as sprint ability and jumping performance (Zagatto, Claus, et al. 2022; Zagatto, Dutra, et al. 2022; de Poli et al. 2020; Wilson et al. 2013). This phenomenon is referred to as “post‐activation performance enhancement” (PAPE) (Blazevich and Babault 2019). Although the underlying mechanisms remain unclear, emerging evidence suggests that PAPE, originally described in anaerobic contexts, may also modulate endurance performance (Boullosa et al. 2018) via integrated neuromuscular (Barnes et al. 2015; Folland et al. 2008; Tillin and Bishop 2009), metabolic (Blazevich and Babault 2019), and hormonal responses (Crewther et al. 2011; Cuenca‐Fernández et al. 2017).

Interestingly, only a limited number of studies have examined the effects of conditioning activity strategies on endurance performance, with most reporting modest improvements in endurance‐related outcomes across different exercise modalities (Boullosa et al. 2020; Chorley and Lamb 2019; Feros et al. 2012; Hancock et al. 2015; Low et al. 2019; Palmer et al. 2009; Silva et al. 2014). However, conditioning activity has not yet been investigated as a strategy to optimize $\dot{V}$O_2peak_ responses, cumulative time spent at ≥ 90% of $\dot{V}$O_2max_, or total exercise duration during HIIT sessions. Although these studies support the potential of PAPE to improve endurance performance, a meta‐analysis conducted by Vasconcelos et al. (2023) reported that the overall effect of PAPE on endurance exercise is very small and supported by low‐quality evidence. Moreover, it was emphasized that the observed significant results largely stemmed from the influence of only two outlier studies. However, these findings do not entirely rule out the possibility that certain conditioning activity strategies, particularly those involving submaximal intensities or short‐duration high‐intensity efforts, may lead to meaningful performance improvements under specific conditions. Protocols designed to elicit the PAPE effect typically require loads ranging from 60% to ≥ 85% of RM (Wilson et al. 2013). Although such loads are often more effective when performed over multiple sets, they may also induce considerable fatigue, which can limit the overall effectiveness of PAPE (Iacono et al. 2019; Wilson et al. 2013).

The effectiveness of the PAPE effect depends on achieving an optimal balance between potentiation and fatigue; sufficient stimulation must be provided without inducing excessive fatigue (Tillin and Bishop 2009). Cluster sets may facilitate post‐exercise recovery by reducing acute neuromuscular fatigue (Páez‐Maldonado et al. 2024; Tufano et al. 2017). Therefore, conditioning activity protocols based on cluster set configurations may be considered an effective strategy for enhancing performance. Particularly in well‐trained athletes who require higher training volumes (Wilson et al. 2013), cluster set configurations are recommended as an effective strategy to minimize fatigue (Tufano et al. 2017) and support acute performance enhancement (Iacono et al. 2019; Nickerson et al. 2018). In addition, this set configuration is proposed to facilitate the clearance of related metabolites (H^+^, Pi, and K^+^) (Haff et al. 2008; Girman et al. 2014; Tufano et al. 2017) and the resynthesis of phosphocreatine, allowing for a greater number of repetitions and helping to maintain performance throughout the set (Tufano et al. 2017). However, the effects of such configurations on PAPE have so far been examined only in anaerobic activities (Iacono et al. 2019; Nickerson et al. 2018, 2019; Boullosa et al. 2013). The current literature does not yet provide conclusive evidence regarding the effects of PAPE protocols involving cluster set configurations on endurance performance such as high‐intensity interval training (HIIT), and this area remains largely underexplored. PAPE protocols combined with cluster set configurations (PAPE_clusterset_) may optimize potentiation effects on HIIT performance more effectively than traditional set configurations (PAPE_traditionalset_) by reducing fatigue. This study aims to investigate the effects of PAPE protocols utilizing different resistance set configurations on $\dot{V}$O_2peak_ responses, cumulative time spent at ≥ 90% $\dot{V}$O_2max_, and total exercise duration during HIIT sessions in well‐trained athletes.

## 2 Materials and Methods

### 2.1 Ethical Approval

The study was approved by the Institutional Review Board of the Gendarmerie and Coast Guard Academy (Protocol no: 220006/6). Experimental procedures were designed according to the rules and principles of the Helsinki Declaration. After explaining the study's procedures, risks, and benefits to all individuals, written informed consent was received from each participant using the approved guidelines and documentation.

### 2.2 Participants

In the G*Power F‐test, a priori analysis conducted to determine the required sample size for the study, an effect size (f) of 0.32, a type I error rate (*α*) of 0.05, a power (1‐*β*) of 0.80, one group, and three measurements were specified. Based on these parameters, the required sample size was calculated as 10. Consequently, 10 well‐trained endurance athletes (age: 23.10 ± 2.02 years; height: 179.32 ± 2.9 cm; body weight: 72.20 ± 3.64 kg; 1RM: 100 ± 8.5 kg; $\dot{V}$O_2max_: 63.3 ± 5.1 mL·kg^−1^ min^−1^; $\dot{V}$O_2max_ velocity: 22.72 ± 1.12 km·h^−1^) participated in the study. All participants regularly engaged in endurance training and actively competed in triathlon or middle‐ and long‐distance running events. In addition, they had experience in resistance training and consistently included high‐intensity back squats and similar exercises in their training programs. The participants had an average of 8 ± 1.29 years of training experience and trained an average of 5.6 ± 0.8 sessions per week. Based on the criteria outlined by De Pauw and colleagues, participants with a relative $\dot{V}$O_2max_ ranging from 55 to 64.9 mL·kg^−1^ min^−1^, coupled with more than 3 years of training history and ≥ 5 h per week, are classified as trained (De Pauw et al. 2013). Participants were instructed to abstain from consuming beverages containing more than 60 mg of caffeine during the measurement period. They were also asked to maintain their regular diet, refrain from eating for at least 2 hours before any test or training session and avoid intense physical activity for at least 24 h prior to testing. None of the participants reported any injuries, medication use, or known systemic diseases (e.g., cardiovascular, pulmonary, metabolic, musculoskeletal, or coronary conditions).

### 2.3 Experimental Protocol

This study employed a randomized crossover design to compare the acute effects of two PAPE protocols, both utilizing the same conditioning activity (half back squat at 85% of 1RM) but differing in set configurations (traditional and cluster sets), as well as a control condition, on HIIT performance. The order of protocol completion was balanced and determined using block randomization (www.random.org). All measurements were conducted under identical conditions at the same facilities. To prevent the potential influence of positive belief and expectancy effects on performance, only participants who had no prior knowledge or experience with PAPE, or prior exercise interventions were included in the study. A pre‐screening questionnaire was used to confirm that participants had no prior knowledge or experience with PAPE or similar performance‐enhancing warm‐up protocols. Additionally, athletes were not informed about the effects of the PAPE protocols either before or during the study. Participants were also not provided with any information regarding the number of repetitions completed or the total exercise duration during the HIIT sessions.

**FIGURE 1:**
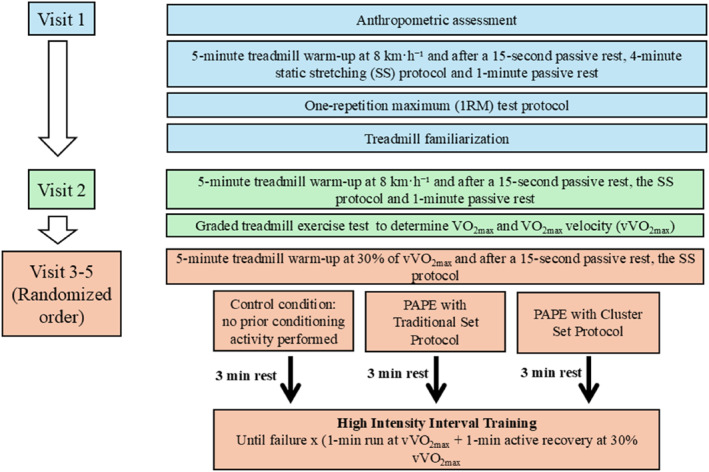
Overview of the experimental design including assessment, familiarization, and intervention sessions across visits 1 to 5 (randomized crossover design).

During the first visit, height and body weight were measured (Seca 767, Germany), and participants performed one‐repetition maximum (1RM) test for half back squat (see below for details on 1RM Determination Protocol for Half Back Squat). To ensure proper technical standardization for the half back squat exercise, a demonstration using an unloaded barbell was provided to each participant, and they were required to perform the movement correctly at least once. Although all participants were familiar with treadmill exercises, a graded treadmill exercise test (GXT) that did not continue to the task of failure was performed to familiarize them with the specific treadmill to be used. This protocol consisted of five stages, each lasting 2 minutes, starting at a speed of 8 km·h^−1^ in the first stage, with the speed increasing by 1 km·h^−1^ at each subsequent stage. During the second visit, participants underwent a GXT to determine their $\dot{V}$O_2max_, velocity at $\dot{V}$O_2max_ ($\dot{V}$O_2max_), and peak heart rate (HR_peak_). On the third, fourth, and fifth visits, participants completed the HIIT protocol in a randomized order following the PAPE_traditionalset_, the PAPE_clusterset_, and control conditions. Prior to each HIIT session, participants completed a five‐minute treadmill run at 30% of $\dot{V}$O_2max_ followed by 4 minutes of static stretching (SS). In all conditions, a 3‐min transition period was applied following the completion of either the control or PAPE protocols (Wilson et al. 2013; Seitz et al. 2014), during which participants remained standing on the treadmill to allow for necessary preparations. Then, the HIIT protocol was initiated. The experimental design is illustrated in Figure 1. Each visit was conducted in a randomized order with a minimum of 48 h and a maximum of 96 h between sessions. To minimize training effects and control for circadian rhythm influences (Souissi et al. 2004), each participant completed all visits within a maximum of 21 days and at the same time of day (± 1 h). All experimental procedures were conducted between 2:00 p.m. and 5:00 p.m. Laboratory conditions were maintained at a temperature of 22°C–24°C and relative humidity of 55%–60%.

### 2.4 Procedures and Data Analysis

#### 2.4.1 Warm‐Up Procedures

Participants completed a 10‐min warm‐up protocol prior to the 1RM test conducted during the first visit and the incremental test protocol conducted during the second visit. The first 5 minutes of this warm‐up consisted of a treadmill exercise at a constant speed of 8 km·h^−1^. Since v$\dot{V}$O _2max_ had not yet been determined at this stage, the warm‐up was standardized in terms of duration and intensity to ensure a submaximal effort. Upon completing the treadmill exercise, participants began the SS protocol following a 15‐s passive rest. The SS protocol included nine lower‐body exercises targeting the following muscle groups: (1) Gastrocnemius, (2) Tibialis anterior, (3) Hamstring muscles, (4) Quadriceps muscle, (5) Gluteus maximus, (6) Iliopsoas, (7) Hip adductors, (8) Hip abductors, and (9) Quadratus lumborum (Çabuk et al. 2025). Stretches 1 and 4 through 9 were performed separately for the right and left legs. Participants were instructed to hold each stretch for 10 s upon reaching their individual pain threshold. A 10‐s passive rest period was provided between each SS exercise. The total duration of the SS protocol was approximately 4 min. One minute after completing the SS protocol, participants began either the 1RM or GXT test.

#### 2.4.2 1 Repetition Maximum Determination Protocol for Half Back Squat

1 RM evaluations were performed using a 20‐kg Olympic barbell (Impex Fitness Products, USA) placed on a squat rack (Fitness Technology, Adelaide, Australia). Following a 5‐min treadmill warm‐up at a fixed speed of 8 km·h^−1^, participants performed two warm‐up sets of 10 repetitions with a light load, approximately 50% of their estimated 1RM. The weight was then incrementally increased to a level where participants could perform three to four repetitions, typically identified within no more than two submaximal attempts. After these attempts, the barbell's weight was further increased by 0.5–5 kg, based on consultation with the participant, until the maximum load that could not be lifted for more than one repetition was determined. A maximum of five 1RM attempts were permitted for each participant. This limit applied only to true maximal attempts and did not include submaximal sets of three to four repetitions intended to estimate the participant's 1RM. Therefore, each participant typically completed up to two submaximal trials in addition to a maximum of five 1RM attempts, resulting in a total of up to seven testing trials per individual.

The half‐squat exercise was performed with free weights until the participants reached 90° knee flexion (thigh parallel to the ground). Rest intervals consisted of 2 min of passive rest between warm‐up sets and 3 min of passive rest between 1RM attempts. Strong verbal encouragement was provided during each maximum attempt to ensure maximal effort. During all trials, trained spotters were positioned on either side of the barbell to ensure safety. All participants used the same squat rack, Olympic barbell, and rubber‐coated weight plates throughout the study. The barbell height was recorded for each participant, and this height was maintained for all load assessments during the study.

### 2.5 Maximal Graded Exercise Test

To determine $\dot{V}$O_2max_, athletes underwent a GXT. The starting velocity for the $\dot{V}$O_2max_ determination test was set at 8 km·h^−1^. After maintaining this velocity for 2 minutes, the running velocity was increased by 1 km·h^−1^ every minute. The incremental velocity increase continued until the participant reached time to task failure. To confirm the incremental test results, at least two of the following criteria were used for termination: (i) heart rate (HR) equal to or greater than 90% of the age‐predicted maximum HR, (ii) a minute‐by‐minute increase in oxygen uptake (VO_2_) of less than 150 mL despite the incremental load, (iii) a respiratory exchange ratio of 1.15 or higher, and (iv) a perceived exertion level (RPE) in the range of 19–20 (Ferretti 2014; Howley et al. 1995). The highest 30‐s moving averages of $\dot{V}$O_2_ and HR were recorded as $\dot{V}$O_2max_ and HR_peak_, respectively. The running velocity reached at the end of GXT was defined as $\dot{V}$O_2max_. The $\dot{V}$O_2max_ was calculated as follows: $\text{Velocity}\;\text{at}\;\text{the}\;\text{last}\;\text{fully}\;\text{completed}\;\text{stage}+\left(\frac{\text{Seconds}\;\text{sustained}\;\text{at}\;\text{the}\;\text{uncompleted}\;\text{stage}}{60\;\text{seconds}}\times 1\;\text{km}{\cdot h}^{-1}\;\right)$ (Adami et al. 2013).

### 2.6 PAPE Protocols

Participants began the half‐squat exercise as soon as they completed the warm‐up protocol. The half‐squat exercise was chosen as it is one of the most frequently used exercises to elicit potentiation responses in the lower extremities (Moir et al. 2011). Participants were instructed to perform half back squats with a controlled tempo of 1 s for the concentric phase and 1 s for the eccentric movements, resulting in a total duration of 2 s per repetition. The total number of repetitions, load, movement velocity, and training volume in the PAPE protocols were matched across the two set configurations. After completing either the cluster or traditional set protocol, two trained spotters provided immediate assistance by supporting the barbell at both ends. This allowed participants to remain in an upright position without bearing the load. As a result, unnecessary exertion was minimized, particularly during the cluster set protocol when compared with the traditional set, and recovery conditions were standardized across both set configurations.

#### 2.6.1 PAPE With Traditional Set Configuration

In the PAPE protocol based on the traditional set configuration, participants performed the half‐squat exercise at 85% of their 1RM for 3 sets of 6 repetitions. Each set was followed by a 120‐s rest period.

#### 2.6.2 PAPE With Cluster Set Configuration

In the PAPE protocol based on the cluster set configuration, participants performed the half‐squat exercise at 85% of their 1RM for 3 sets of 2 + 2 + 2 repetitions. A 20‐s rest period was provided between each pair of repetitions, and an 80‐s rest period was provided between sets.

#### 2.6.3 HIIT Protocol

The HIIT protocol involved 1‐min running intervals at the velocity of the final stage reached during the GXT, followed by 1‐min active recovery periods at 30% of their $\dot{V}$O_2max_ (maintaining a 1:1 work‐to‐rest ratio. Participants continued the work bouts for as many repetitions as they could maintain. To confirm that participants reached task failure during the HIIT protocol, several criteria were monitored throughout the session. These included: (1) reaching a score of 19 or 20 on the RPE scale, (2) failure to maintain the target running speed during the work intervals, and (3) voluntary termination of the session due to perceived maximal effort. The HIIT protocol was performed three times on separate days, with each session corresponding to one of the following conditions: after the standardized warm‐up, after the PAPE_traditionalset_, and after the PAPE_clusterset_.

### 2.7 Physiological Responses

During the test, participants breathed through a low‐volume (90 mL) low‐resistance (5.5 cm H_2_O at 510 L/min) face mask with a turbine assembly. Gas samples were continuously transferred from the mask via a 2 m long sampling line with an internal diameter of 0.5 mm to a breath‐by‐breath gas analyzer (Fitmate Pro, Cosmed, Rome, Italy). The device uses a turbine flowmeter to measure ventilation and a galvanic fuel cell oxygen sensor to analyze the fraction of oxygen in expired gases. It is also equipped with sensors that measure humidity, temperature, and barometric pressure, which are used in the device's internal calculations. All procedures were carried out as described by Nieman et al. (2007).

Capillary blood lactate [La^−^] concentrations were measured using fingertip samples. Briefly, the fingertip skin was punctured using an Accu‐Chek disposable lancet (Roche Diagnostics, West Sussex, UK), and gentle pressure was applied to the finger to extract blood. The first drop was discarded, and the second drop was collected for [La^−^] analysis. Blood samples were obtained 1 minute before the start of HIIT ([La_preHIIT_]), and at 1, 3, and 5 min after the completion of the HIIT protocol. [La^−^] levels were analyzed using a portable [La^−^] analyzer (H/Cosmos, Germany). The highest blood [La^−^] value obtained after completing any HIIT protocol was recorded as the maximal blood [La^−^] concentration ([La_max_]). The difference between [La_max_] and resting blood [La^−^] concentration was recorded as delta blood [La^−^]. Additionally, a 15‐point Borg scale (i.e., 6–20) was used to measure the RPE following each HIIT protocol.

### 2.8 Calculation of Physiological Responses

For each HIIT protocol, the highest values of $\dot{V}$O_2_ and HR were calculated based on a 30‐s moving average and were referred to as $\dot{V}$O_2peak_ and HR_peak_, respectively. The total time spent at ≥ 90% $\dot{V}$O_2max_ during HIIT was calculated by summing all 5‐s intervals where $\dot{V}$O_2_ met or exceeded 90% of $\dot{V}$O_2max_ (Norouzi et al. 2023).

### 2.9 Statistical Analysis

The normality of the data was evaluated with the Shapiro‐Wilk test. Appropriate statistical tests were selected based on the results of the normality. Differences among variables with a normal distribution ($\dot{V}$O_2max_, $\dot{V}$O_2peak_, HR_peak_, [La_max_]) were examined using repeated measures ANOVA with Bonferroni correction. Differences in variables [La_preHIIT_], time spent at ≥ 90% of $\dot{V}$O_2max_, and total exercise duration not showing a normal distribution were analyzed using the Friedman test. Pairwise comparisons for these variables were conducted using the Wilcoxon signed‐rank test. To control Type I error due to multiple comparisons, the Bonferroni correction was applied, and the significance threshold was adjusted to *p* < 0.017 (0.05 divided by 3 pairwise comparisons). To compare the magnitude of the between‐group differences, partial eta squared (*η* ^2^_p_) values were calculated for ANOVA comparisons, with effect sizes classified as small (≥ 0.01), medium (≥ 0.06), or large (≥ 0.14) (Cohen 1988). Additionally, effect size of the differences in pairwise comparisons were calculated according to Cohen's d coefficient (Cohen 1998). Cohen's d was categorized as trivial effect (< 0.2), small effect (0.2–0.5), medium effect (0.5–0.8), and large effect (> 0.8). Statistical significance was accepted at *p* < 0.05. Results are reported as mean ± SD.

## 3 Results

### 3.1 Peak Oxygen Uptake Between the HIIT Protocols and Maximal Oxygen Consumption Obtained From GXT

Although the ANOVA indicated a marginally significant difference among conditions (*F*(1.6,14.5) = 4.03; *p* = 0.048; *η* ^2^_p_ = 0.31), the pairwise post hoc comparisons did not reveal any statistically significant differences (*p* = 0.123–0.566; Cohen's d = 0.50–0.88).

### 3.2 Peak Oxygen Uptake Among the HIIT Protocols

**FIGURE 2:**
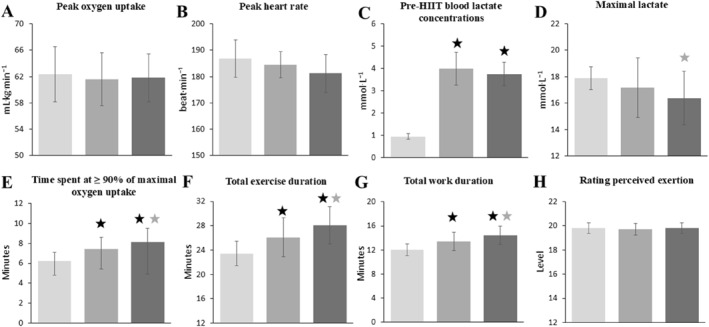
Physiological and time responses to the three HIIT conditions are shown in the figure as means with standard deviations. A; Peak oxygen consumption response obtained during HIIT, B; Time spent at ≥ 90% of $\dot{V}$O_2max_ during HIIT, C; The maximal blood lactate response obtained after HIIT, D; The total exercise duration of HIIT, 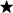; significantly different from PAPE_control_, 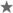; significant difference between PAPE_traditionalset_ and PAPE_clusterset_. HIIT; High intensity interval training, PAPE_traditionalset_; post‐activation performance enhancement with traditional set configuration, PAPE_clusterset_; Post‐activation performance enhancement with cluster set configuration. The three shades of gray indicate the three HIIT conditions applied in a randomized crossover design: light gray = Control, medium gray = PAPE_traditionalset_, and dark gray = PAPE_clusterset_.

There were no significant differences in $\dot{V}$O_2peak_ responses among the HIIT protocols (*F*(2,18) = 1.8; *p* = 0.198; *η* ^2^_p_ = 0.16) (Figure 2). Among the three pairwise comparisons, the *p*‐values ranged from 0.225 to 1, and Cohen's d values ranged from 0.14 to 0.63.

### 3.3 Peak Oxygen Uptake Responses During Work Bout Periods

**FIGURE 3:**
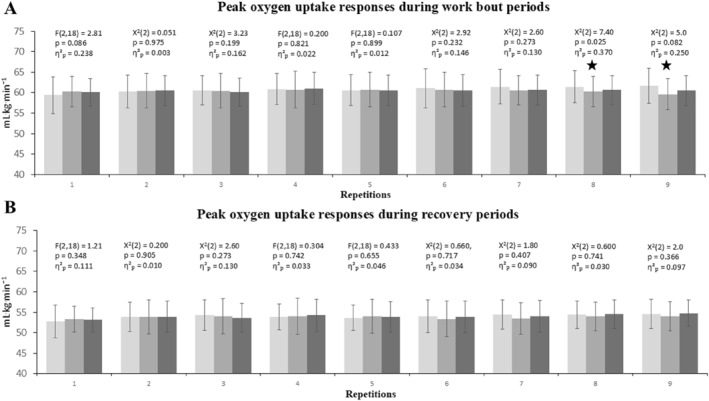
Peak oxygen uptake ($\dot{V}$O_2peak_) responses during each repetition of the three different HIIT protocols. (A) The upper panel shows $\dot{V}$O_2peak_ responses during the work bout periods, (B) the lower panel presents the responses during the subsequent recovery periods. Each bar represents the mean ± standard deviation for each repetition (1–9). 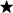; significantly different from PAPE_control_. HIIT; High intensity interval training, PAPE_traditionalset_; post‐activation performance enhancement with traditional set configuration, PAPE_clusterset_; Post‐activation performance enhancement with cluster set configuration. The three shades of gray indicate the three HIIT conditions applied in a randomized crossover design: light gray = Control, medium gray = PAPE_traditionalset_, and dark gray = PAPE_clusterset_.

Among the work bouts, a significant difference between conditions was found only in the eighth interval (*χ* ^2^(2) = 7.40, *p* = 0.025, *η* ^2^_p_ = 0.370), according to the Friedman test (Figure 3). Pairwise comparisons using the Wilcoxon test indicated that this difference originated from the comparison between the control and PAPE_traditionalset_ conditions. No statistically significant differences were observed in the comparisons between the control and PAPE_clusterset_ (*p* = 0.053; Cohen's d = 0.61) or between the PAPE_traditionalset_ and PAPE_clusterset_ conditions (*p* = 0.414; Cohen's d = 0.26).

### 3.4 Peak Oxygen Uptake Responses During Recovery Periods

Throughout the recovery periods, neither ANOVA (*F*(2,18) = 0.304–1.21, *p* = 0.348–0.742, *η* ^2^_p_ = 0.033–0.111) nor Friedman tests (*χ* ^2^(2) = 0.200–2.60, *p* = 0.273–0.905, *η* ^2^_p_ = 0.010–0.130) (Figure 3), nor any of the multiple comparisons revealed any statistically significant differences between the conditions (non‐parametric tests: *p* = 0.028–0.96; Cohen's d = 0.016–0.69; parametric tests: *p* = 0.017–0.84; Cohen's d = 0.018–0.47).

### 3.5 Time Spent at ≥ 90% of Maximal Oxygen Uptake Among the HIIT Protocols

There were significant differences in the time spent at ≥ 90% of $\dot{V}$O_2max_ across the HIIT protocols (*χ* ^2^(2) = 14.6; *p* < 0.001). Time spent at ≥ 90% of $\dot{V}$O_2max_ was significantly lower for HIIT following the control condition, compared with HIIT following the PAPE_traditionalset_ (*p* < 0.001; *z* = −2.81; Cohen's d = 0.89) and HIIT following the PAPE_clusterset_ (*p* < 0.001; *z* = −2.84; Cohen's d = 0.90), with differences observed between HIIT following the PAPE_traditionalset_ and HIIT following the PAPE_clusterset_ (*p* < 0.001; *z* = −2.77; Cohen's d = 0.88) (Figure 2).

### 3.6 Time Spent at ≥ 90% of Maximal Oxygen Uptake During Work Bout Periods in HIIT Conditions

**FIGURE 4:**
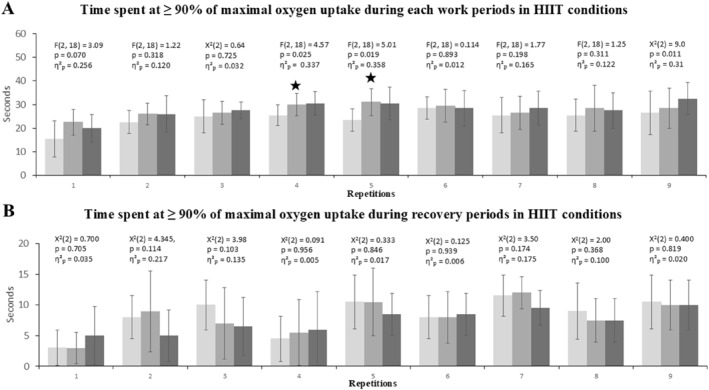
Time spent at ≥ 90% of maximal oxygen uptake ($\dot{V}$O_2max_) during each repetition of the three different HIIT protocols. (A) The upper panel shows the time spent at ≥ 90% of $\dot{V}$O_2max_ during the work bout periods, (B) the lower panel presents the time spent at ≥ 90% of $\dot{V}$O_2max_ during the subsequent recovery periods. Each bar represents the mean ± standard deviation for each repetition (1–9). 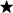; significantly different from PAPE_control_. HIIT; High intensity interval training, PAPE_traditionalset_; post‐activation performance enhancement with traditional set configuration, PAPE_clusterset_; Post‐activation performance enhancement with cluster set configuration. The three shades of gray indicate the three HIIT conditions applied in a randomized crossover design: light gray = Control, medium gray = PAPE_traditionalset_, and dark gray = PAPE_clusterset_.

A significant difference was observed between the three HIIT conditions during the fourth (*F*(2, 18) = 4.57; *p* = 0.025; *η* ^2^_p_ = 0.337), fifth (*F*(2, 18) = 5.01; *p* = 0.019; *η* ^2^_p_ = 0.358), and ninth (*χ* ^2^(2) = 9.0; *p* = 0.011; *η* ^2^_p_ = 0.310) work bouts (Figure 4). Post hoc pairwise comparisons revealed that this difference occurred only between the PAPE_traditionalset_ and control conditions in the fourth (PAPE_traditionalset_ vs. Control: *p* = 0.030, Cohen's d = 1.02; PAPE_clusterset_ vs. Control: *p* = 0.096, Cohen's d = 0.80; PAPE_traditionalset_ vs. PAPE_clusterset_: *p* = 1.00, Cohen's d = 0.07) and fifth intervals (PAPE_traditionalset_ vs. Control: *p* = 0.045, Cohen's d = 0.95; PAPE_clusterset_ vs. Control: *p* = 0.103, Cohen's d = 0.79; PAPE_traditionalset_ vs. PAPE_clusterset_: *p* = 1.00, Cohen's d = 0.06). Although the Friedman test indicated a significant overall effect between conditions during the ninth interval, the Wilcoxon pairwise comparisons did not yield statistically significant differences (*p* = 0.026–0.109; Cohen's d = 0.51–0.70).

### 3.7 Time Spent at ≥ 90% of Maximal Oxygen Uptake During Recovery Periods in HIIT Conditions

During the recovery periods, neither the ANOVA (*χ* ^2^(2) = 0.09–4.34, *p* = 0.956–0.114, *η* ^2^_p_ = 0.005–0.217) nor the post hoc comparisons revealed any significant differences between conditions (*p* = 0.063–1.0, *z* = −1.85–1.0, Cohen's d = 0.58–1.0) (Figure 4).

### 3.8 The Ratio of Time Spent at ≥ 90% of the Maximal Oxygen Uptake Attained During the Work Periods to the Total Work Duration

**FIGURE 5:**
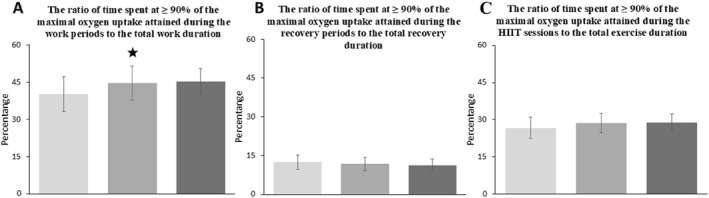
Relative time spent at ≥ 90% of $\dot{V}$O_2max_. A; The left section of the figure shows the relative time spent at ≥ 90% of $\dot{V}$O_2max_ during total work duration, whereas the middle section shows the relative time spent at ≥ 90% of $\dot{V}$O_2max_ during recovery periods. The right section illustrates the relative time spent at ≥ 90% of $\dot{V}$O_2max_ during total exercise duration (work bout + recovery). Bars represent mean ± standard deviation. 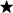; significantly different from PAPE_control_. HIIT; High intensity interval training, PAPE_traditionalset_; post‐activation performance enhancement with traditional set configuration, PAPE_clusterset_; Post‐activation performance enhancement with cluster set configuration. The three shades of gray indicate the three HIIT conditions applied in a randomized crossover design: light gray = Control, medium gray = PAPE_traditionalset_, and dark gray = PAPE_clusterset_.

In the HIIT conditions, a significant difference was observed only in the proportion of time spent at or above 90% of $\dot{V}$O_2max_ relative to the total work duration (*F*(2,18) = 3.93, *p* = 0.038, *η* ^2^_p_ = 0.304) (Figure 5). Although both the PAPE_traditionalset_ (*p* = 0.04, *t* = 4.62, Cohen's d = 1.46) and PAPE_clusterset_ (*p* = 0.235, *t* = 1.98, Cohen's d = 0.63) conditions showed higher percentages compared with the control condition, post hoc analyses revealed a statistically significant difference only between the PAPE_traditionalset_ and the control group. On the other hand, no significant difference was found between the PAPE_traditionalset_ and PAPE_clusterset_ conditions (*p* = 1.0, *t* = −0.36, Cohen's d = 0.11).

### 3.9 The Ratio of Time Spent at ≥ 90% of the Maximal Oxygen Uptake Attained During the Recovery Periods to the Total Recovery Duration

No significant difference was found between the HIIT conditions in terms of the proportion of time spent at or above 90% of $\dot{V}$O_2max_ during the recovery phases relative to the total recovery duration (*F*(2,18) = 0.724, *p* = 0.499, *η* ^2^_p_ = 0.074) (Figure 5). In the three pairwise comparisons conducted, *p*‐values ranged from 0.934 to 1, and Cohen’s d values ranged from 0.18 to 0.34.

### 3.10 The Ratio of Time Spent at ≥ 90% of the Maximal Oxygen Uptake Attained During the HIIT Sessions to the Total Exercise Duration

No significant difference was observed between the HIIT conditions in terms of the proportion of time spent at or above 90% of $\dot{V}$O_2max_ relative to the total exercise duration (*F*(2,18) = 1.57, *p* = 0.235, *η* ^2^_p_ = 0.149) (Figure 5). According to post hoc analyses, although the *p*‐values for the comparisons of the control condition with the PAPE_traditionalset_ and PAPE_clusterset_ conditions were 0.131 and 0.778, respectively, Cohen's d values were reported as 0.78 and 0.38, respectively.

### 3.11 Total Exercise Duration and Total Work Duration at HIIT Among the HIIT Protocols

There were significant differences in the total exercise duration across the HIIT protocols (*F*(2,18) = 23.9, *p* < 0.001, *η* ^2^_p_ = 0.719) (Figure 2). The total exercise duration during HIIT was significantly longer following both the PAPE_traditionalset_ (*p* = 0.006, *t* = −4.24; Cohen's d = 1.34) and the PAPE_clusterset_ (*p* = 0.001, *t* = −5.67; Cohen's d = 1.80) compared with the control condition. The HIIT session following the PAPE_clusterset_ resulted in a significantly longer exercise duration than that following the PAPE_traditionalset_ (*p* = 0.026, *t* = −3.34; Cohen's d = 1.05) (Figure 2).

Similarly, significant differences in total work duration were observed across the HIIT protocols (*F*(2,18) = 31.9, *p* < 0.001, *η* ^2^_p_ = 0.714) (Figure 2). During the HIIT sessions, total work duration was significantly longer following both the PAPE_traditionalset_ (*p* = 0.005; *t* = −4.42; Cohen's d = 1.40) and the PAPE_clusterset_ (*p* = 0.001; *t* = −5.70; Cohen's d = 1.80) protocols compared with the control condition. Furthermore, the HIIT session performed after the PAPE_clusterset_ protocol resulted in a significantly longer total work duration than the session following the PAPE_traditionalset_ (*p* = 0.043; *t* = −3.02; Cohen's d = 0.95) (Figure 2).

### 3.12 Lactate Responses Among the HIIT Protocols

There were significant differences in [La_preHIIT_] responses among the conditions (*F*(2, 18) = 193; *p* < 0.001; *η* ^2^_p_ = 0.955) (Figure 2). Compared with the control condition, [La_preHIIT_] responses were significantly higher following both the PAPE_traditionalset_ (*p* < 0.001, *t* = −13.3, Cohen's d = 4.20) and the PAPE_clusterset_ (*p* < 0.001, *t* = −18.4, Cohen's d = 5.80). Additionally, [La_preHIIT_] responses following the PAPE_clusterset_ were similar to those observed following the PAPE_traditionalset_, with no statistically significant difference between the two conditions (*p* = 0.198, *t* = −2.10, Cohen's d = 0.66) (Figure 2). Also, there were no significant differences in [La_max_] responses among the HIIT protocols (*F*(2, 18) = 2.81; *p* = 0.086; *η* ^2^_p_ = 0.238) (Figure 2). Although the main effect of condition did not reach statistical significance in the ANOVA, post hoc analysis revealed that the PAPE_traditionalset_ elicited marginally but statistically higher [La_max_] responses compared with the PAPE_clusterset_ following the HIIT session (*p* = 0.049, *t* = 2.94, Cohen's d = 0.93). [La_max_] responses in the control condition did not differ from those observed in either the PAPE_traditionalset_ (*p* = 1.0, *t* = 0.90, Cohen's d = 0.29) or the PAPE_clusterset_ conditions (*p* = 0.184, *t* = 2.13, Cohen's d = 0.67).

### 3.13 Peak Heart Rate Responses Among the HIIT Protocols

There were no significant differences in HR_peak_ responses among the HIIT protocols (*F*(2,18) = 2.41; *p* = 0.118; *η* ^2^_p_ = 0.21) (Figure 2). Among the three pairwise comparisons, the *p*‐values ranged from 0.258 to 1, and the effect sizes ranged from 0.28 to 0.60.

### 3.14 Rating Perceived Exertion Level Among the HIIT Protocols

There were no significant differences in the RPE levels across the HIIT protocols (*χ* ^2^(2) = 0.33, *p* = 0.846) (Figure 2). Among the three pairwise comparisons, the *p*‐values ranged from 0.564 to 1, and the effect sizes ranged from 0 to 0.18.

## 4 Discussion

The aim of this study was to evaluate the effects of the PAPE protocols induced by high‐intensity strength exercises on total exercise duration, higher $\dot{V}$O_2peak_ responses, and time spent at ≥ 90% of $\dot{V}$O_2max_ during HIIT sessions. The main findings indicate that PAPE applications did not meaningfully alter $\dot{V}$O_2peak_ responses during HIIT, with these responses remaining comparable across conditions and consistent with the $\dot{V}$O_2max_ values obtained from the GXT. Similarly, no marked differences were observed among HIIT conditions in [La^−^], HR_peak_, or RPE responses. In contrast, both traditional‐ and cluster set‐based PAPE protocols enhanced exercise tolerance, resulting in a longer duration spent at ≥ 90% of $\dot{V}$O_2max_. Notably, the more pronounced improvements observed following the cluster set configuration suggest that the effectiveness of PAPE applied before HIIT may depend on the resistance exercise set structure.

These observations further suggest that the ergogenic effects of PAPE during HIIT are unlikely to be mediated by changes in maximal physiological responses, but rather by alterations in the temporal characteristics of the $\dot{V}$O_2_ response during repeated work bouts. In support of this interpretation, Marinari et al. (2024) demonstrated that a heavy‐intensity priming exercise reduced the time required to reach the $\dot{V}$O_2max_ breakpoint and increased the time spent at $\dot{V}$O_2max_ (i.e., prolonged the $\dot{V}$O_2max plateau_) during ramp‐incremental exercise, while also increasing peak power output without altering $\dot{V}$O_2max_. These effects were primarily attributed to faster $\dot{V}$O_2_ kinetics (Marinari et al. 2024). Consistent with this mechanistic framework, both traditional‐ and cluster set‐based PAPE protocols in the present study enhanced exercise tolerance and increased time spent at ≥ 90% of $\dot{V}$O_2max_, despite comparable $\dot{V}$O_2peak_, blood [La^−^], HR_peak_, and RPE responses across conditions. Collectively, these findings indicate that priming strategies, including resistance‐based PAPE interventions, may enhance high‐intensity exercise performance predominantly by accelerating $\dot{V}$O_2_ kinetics and facilitating an earlier attainment of high fractions of $\dot{V}$O_2max_, rather than by increasing maximal cardiorespiratory capacity. Notably, although the PAPE_traditionalset_ and PAPE_clusterset_ conditions exhibited longer total work durations than the control condition by 84 s (+ 1.4 repetitions) and 144 s (+ 2.4 repetitions), respectively, the corresponding increases in time spent at ≥ 90% of $\dot{V}$O_2max_ were 73 and 90 s. This indicates that time spent at ≥ 90% of $\dot{V}$O_2max_ does not increase in direct proportion to total exercise duration and suggests that $\dot{V}$O_2_ kinetics may have been accelerated during HIIT repetitions following the PAPE interventions. The findings regarding the ratio of time spent at ≥ 90% of $\dot{V}$O_2max_ during only the work periods of the HIIT sessions to the total work duration support our hypothesis. In the control condition, participants spent 40.3% of the total work duration at ≥ 90% of $\dot{V}$O_2max_, whereas this ratio was 44.7% and 45.4% for the PAPE_traditionalset_ and PAPE_clusterset_ protocols, respectively. In addition, during the fourth and fifth work bouts of the HIIT sessions, both the PAPE_traditionalset_ and PAPE_clusterset_ protocols demonstrated statistically significant increases in the time spent at ≥ 90% of $\dot{V}$O_2max_ compared with the control condition (Figure 4A). Although no statistically significant differences were observed during the earlier work bouts, medium to large *η* ^2^_p_ values observed across repetitions (with the exception of the third bout) (Figure 4A) suggest that the PAPE protocols may generally shorten the time required to reach ≥ 90% of $\dot{V}$O_2max_.

Although the exact mechanisms responsible for PAPE‐induced improvements in endurance performance remain to be fully elucidated, the present findings may be partially explained by acute physiological changes based on previous literature. These include transient increases in intramuscular temperature (Blazevich and Babault 2019) and fluid content (Blazevich and Babault 2019), potential modulation of spinal reflex excitability (e.g., H‐reflex) (Folland et al. 2008), greater recruitment of motor units (Tillin and Bishop 2009), acute hormonal responses (e.g., testosterone and catecholamines) (Crewther et al. 2011; Cuenca‐Fernández et al. 2017), and increased stiffness of the muscle‐tendon unit (Blazevich and Babault 2019; Barnes et al. 2015). Through these mechanisms, the PAPE conditions may have facilitated faster $\dot{V}$O_2_ kinetics during HIIT repetitions, allowing participants to reach ≥ 90% of $\dot{V}$O_2max_ more quickly. Although these mechanisms provide a plausible physiological explanation for the observed performance enhancements, placing the present findings within the context of existing literature remains challenging, as no prior research has specifically examined the effects of any conditioning activity followed by HIIT training. Although direct similar studies are absent, limited research has explored the immediate effects of conditioning activities on endurance performance (Boullosa et al. 2020; Feros et al. 2012; Low et al. 2019; Silva et al. 2014). Nevertheless, the limited available evidence indicates that acute PAPE protocols may improve endurance performance by enhancing exercise tolerance, without substantially altering maximal physiological responses such as $\dot{V}$O_2peak_, blood [La^−^], HR_peak_, or RPE (Gama et al. 2023; Nieman et al. 2007; Silva et al. 2014). However, the reported findings remain inconsistent, and PAPE effects appear to be highly dependent on methodological factors, including the resistance exercise protocol used, recovery duration, warm‐up standardization, and the balance between potentiation and fatigue (Boullosa et al. 2018, 2020; Feros et al. 2012; Low et al. 2019; Wilson et al. 2013). Notably, the absence of significant performance improvements following PAPE in some studies has been attributed to elevated blood [La^−^] levels at the start of testing, suggesting an inadequate balance between potentiation and fatigue (Chorley and Lamb 2019). Previous studies have indicated that pre‐exercise protocols that do not alter blood [La^−^] levels do not influence $\dot{V}$O_2_ kinetics or exercise tolerance, whereas those that elevate blood [La^−^] above 5–6 mmol·L^−1^ may affect $\dot{V}$O_2_ kinetics but do not improve, and may even reduce, exercise tolerance (Bailey et al. 2009; Wilkerson et al. 2004; Ferguson et al. 2007). Furthermore, Burnley et al. (2005) reported that high‐intensity exercise performed prior to the main exercise bout could lead to elevated blood [La^−^] levels and prematurely reduce anaerobic distance capacity (D′), thereby shortening time to task failure. They also noted that initial blood [La^−^] levels in the range of 3–5 mmol·L^−1^ could be associated with improved performance once muscle homeostasis is re‐established (Burnley et al. 2005; Bailey et al. 2009; Miura et al. 2009; Jones et al. 2003). In contrast, studies have shown that when baseline blood [La^−^] concentrations fall below 2 mmol·L^−1^, the influence of prior conditioning activity on $\dot{V}$O_2_ kinetics tends to disappear (Bailey et al. 2009; Burnley et al. 2005). Similarly, the interaction between the PAPE protocols applied before HIIT and initial blood [La^−^] levels in our study is noteworthy. In our research, initial [La^−^] responses before HIIT in the PAPE_clusterset_ and the PAPE_traditionalset_ conditions were at levels of 3.47 and 3.09 mmol.L^−1^, respectively, whereas the control group had a level of 1.03 mmol.L^−1^. Numerous studies have indicated that a moderate increase in [La^−^] is associated with improved subsequent endurance performance (Jones et al. 2003; Palmer et al. 2009; Burnley et al. 2005; Marinari et al. 2024, 2025) possibly providing a protective effect against fatigue. Further studies have observed that heavy or severe exercise causing a moderate increase in [La^−^] accelerates $\dot{V}$O_2_ kinetics, aiding in a faster transition from anaerobic to aerobic energy systems at the outset. Taken together, these findings highlight the importance of carefully controlling the intensity and duration of pre‐conditioning activity, as well as the recovery time prior to competition.

Previous research emphasizes that PAPE protocols implemented without adequate recovery may impair performance rather than enhance it, whereas excessively long recovery periods may reduce fatigue but also abolish the potentiation effect (Boullosa et al. 2018; Tillin and Bishop 2009). For this reason, PAPE protocols may be considered a “double‐edged sword.” Although optimal recovery durations are generally suggested to fall within the range of 8–12 min (Gouvea et al. 2013), this timing may vary depending on the intensity and structure of the conditioning protocol (Parry et al. 2008; Ingham et al. 2013; Bailey et al. 2009). In the present study, however, despite implementing only a 3‐min recovery period following squat‐based PAPE protocols, time to task failure during HIIT was 20% and 25% longer in the two PAPE conditions compared with the control condition, respectively. Both the PAPE_traditionalset_ and PAPE_clusterset_ protocols resulted in an increased time spent at ≥ 90% of $\dot{V}$O_2max_ during the HIIT session, with this effect being more pronounced following the PAPE_clusterset_ protocol. Although both PAPE protocols were applied using the same volume and intensity, the blood [La^−^] response observed after the PAPE_clusterset_ protocol showed a borderline statistically significant difference (*p* = 0.051). Nevertheless, it would be overly assertive to claim the superiority of the PAPE_clusterset_ protocol over the PAPE_traditionalset_ protocol based solely on this small but statistically significant difference in [La^−^] concentration (0.39 mmol·L^−1^).

The cluster set method involves dividing the repetitions of a set into smaller groups separated by short rest intervals (Tufano et al. 2017; Cin et al. 2021). This set configuration may facilitate the clearance of metabolites (H^+^, Pi, and K^+^) and phosphocreatine resynthesis, thereby helping to maintain performance throughout the set and allowing a greater total training volume to be completed (Haff et al. 2008; Girman et al. 2014; Oliver et al. 2015). In the present study, the lower blood [La^−^] levels observed prior to HIIT following the PAPE_clusterset_ protocol suggest that this set structure induced lower metabolic stress and that athletes may have initiated HIIT with a higher D′ level. These findings indicate that a cluster set‐based PAPE protocol may represent a feasible strategy for endurance athletes. In the existing literature, post‐2019 studies have primarily examined PAPE protocols within the context of anaerobic performance and have compared traditional and cluster set structures largely within this framework. From this perspective, the present study represents one of the limited investigations addressing the effects of a cluster set‐based PAPE protocol on HIIT effectiveness and endurance performance. Notably, a meta‐analysis by Jukic et al. (2020) demonstrated that cluster set configurations are associated with lower [La^−^] accumulation compared with traditional sets. Similarly, Páez‐Maldonado et al. (2024) reported that cluster sets reduce metabolic stress while better preserving mechanical performance. Taken together, these findings suggest that the cluster set configuration may support a more favorable balance between potentiation and fatigue. Although much of the available evidence is derived from anaerobic exercise contexts, the present results indicate that PAPE protocols implemented with cluster sets may also be effective during endurance‐based HIIT sessions.

## 5 Conclusion

The effects of conditioning activity strategies on endurance have been examined in a limited number of studies, and these studies have primarily focused on endurance test performance or competition outcomes (Boullosa et al. 2020; Chorley and Lamb 2019; Feros et al. 2012; Hancock et al. 2015; Silva et al. 2014). To the best of our knowledge, this is the first study to investigate the effects of PAPE protocols on HIIT. Our findings demonstrate that both traditional and cluster set‐based PAPE protocols increase total exercise duration, total work time, and the time spent at ≥ 90% of $\dot{V}$O_2max_ during HIIT sessions. These increases were especially more pronounced in the cluster set condition. Although both PAPE protocols positively influenced HIIT effectiveness and performance, the cluster set‐based approach may provide a stronger physiological stimulus, making it a more effective method for enhancing the efficiency of HIIT protocols.

## Author Contributions

M.C.: data collection, conception or design of the work, data analysis and interpretation, drafting the article, critical revision of the article. O.S.: conception or design of the work, drafting the article, critical revision of the article. R.C.: conception or design of the work, data analysis and interpretation, drafting the article, critical revision of the article. All authors read and approved the final manuscript.

## Funding

The authors have nothing to report.

## Ethics Statement

The study was approved by the Clinical Research Ethics Committee of Muğla Sıtkı Koçman University (Protocol no: 220006/6).

## Conflicts of Interest

The authors do not have any conflicts of interest.

## Data Availability

Data generated and/or analyzed during this study are available from the corresponding author upon reasonable request.
